# Qualitative ultrastructural analysis of the submandibular salivary glands after administration of khat: in vivo study

**DOI:** 10.1186/s13104-021-05595-8

**Published:** 2021-05-13

**Authors:** Gamilah Al-Qadhi, Rabab Mubarak

**Affiliations:** 1 Department of Basic Dental Sciences, Faculty of Dentistry, University of Science and Technology, Taiz, Yemen; 2 Department of Oral Biology, Faculty of Dentistry, Cairo University, Cairo, 11553 Egypt

**Keywords:** Khat, Catha edulis, Submandibular salivary gland, Ultrastructural changes, TEM

## Abstract

**Objective:**

Khat (*Catha edulis* Forssk) plant has been widely chewed for its psychostimulatory effects in the African and Arabian Peninsula, particularly in Yemen. Considering the khat leaves are gradually chewed without swallowing, while its active constituents are extracted into saliva, studying the effect of khat on salivary glands is necessary. This work is an extension of the previously published work that studied the effect of khat extract on the rats' submandibular salivary glands in terms of histological and immunohistochemical evaluations. The current research note aimed to better understand this effect on the ultrastructure of submandibular salivary gland cells by using transmission electron microscope.

**Results:**

Oral administration of khat extract produced degenerative changes in the secretory and ductal cells of rats' submandibular salivary glands. These changes involved irregular boundaries of variable sized-nuclei, dilated RER, cytoplasmic vacuoles as well as swollen and degenerated mitochondria.

**Supplementary Information:**

The online version contains supplementary material available at 10.1186/s13104-021-05595-8.

## Introduction

Khat or qat (Catha edulis Forssk) is a type of plant known for its psychostimulatory effects. Khat chewing is a common bad social habit among African and Arabian people, particularly Yemeni ones. People gradually chew khat for several hours every day and kept the khat bolus inside the lower buccal vestibule to make a characteristic swelling on one side of the cheek. Later on, the leaves are spit out and the obtained juice is swollen [1, 2].

The active constitutes of khat, including (−) cathinone, which has a similar pharmacological picture as amphetamine, and cathine [3]. Various studies have demonstrated the negative effects of khat on body, such as the cardiovascular system [4, 5], the central nervous system [6, 7], neonatal, maternal health [8, 9], fertility [10, 11], cellular toxicity, apoptosis in human cells [12–14], structural chromosomal aberrations [15], and increased production of reactive oxygen species (ROS) [16].

An association between khat chewing and oral lesions has been illustrated in several articles, including white lesions, periodontal diseases, and tooth discoloration, where they were significantly more prevalent in the khat chewers than non-chewers [17–20]. More importantly, prolonged exposure to the khat may be a contributing factor for the high incidence of oral squamous carcinoma [21].

Saliva has a critical role in the preservation and maintenance of oral health and dental integrity. Dysfunction of saliva occurs as a sequence of a certain disease or as a side effect of several drugs or even as a result of bad habits [22]. Oral dryness was reported in khat chewers 30 min after the beginning of the khat‐chewing session [20].

Similarly, decreased salivary parameters such as the flow rate and pH were observed among khat chewers and this condition might be related to the amphetamine‐like effect of khat or continuous mechanical action of the salivary glands during the chewing session [23]. Histological analysis showed signs of atrophied acini and degenerated ductal cells in rat's salivary glands treated by khat [24]. It should be mentioned that this research note is considered as an extension of that previous work [24], which was published in a local college journal, and aimed to better understand the effects of khat extract on the submandibular salivary glands in terms of ultrastructural profile.

## Main text

### Materials and methods

#### Experimental design

Rats are reliable models to study the salivary glands as they have considerable morphological and histological similarities with the human one [25]. Healthy adult male Albino Wistar rats (species: Rattus Norvegicus) (n = 20) of average weight 150 ± 10 g and 3–4 months old were used in this study. The rats were obtained from and housed in the animal house at the Institute of Ophthalmology, Cairo University under the control of a specialized veterinarian. All animals were kept under the same housing and feeding conditions (12–12 h light/dark cycle, 25 ± 2 °C room temperature, and 50 ± 5% relative humidity).

They were housed in a specially designed filter-top polypropylene cages (48.5 cm L × 33 cm W × 21 cm H). The cages were cleaned twice weekly, bedded with woodchips, and supplied with cardboard boxes, and fleece chew toy for environmental enrichment. They were fed with the standard laboratory rat diet (Meladco Feed Co., Cairo, Egypt) and water ad libitum*.* The experiment was conducted after one week of acclimatization of animals to their new environment in the research room.

Each rat was matched with a specific number (from 1 to 20) by the technician at the animal house and randomly divided into two groups (10 rats/group) using the randomizer website. Sample size calculation was carried out using resources equation method as the current study didn’t have any quantitative data, so 10 and 20 rats can be considered as an adequate sample, in which E measured by following formula: E = total number of animals—total number of groups [26].

The control group received 1 ml of normal saline, and the khat-treated group received 1000 mg/kg b.w (= 1 ml) of aqueous khat extract twice a week (Monday and Thursday) for ten consecutive weeks via a sterile oral gavage flexible polyurethane-feeding tube. The dose was considered as a medium dose and selected according to previous reports [15, 27]. Among different types of khat, the Nehmi khat leaves had the highest concentration of cathinone and tannic acid [28]. Therefore, they were used to extract the desired dose following the previous work [29].

#### Transmission electron microscopy (TEM)

After ten weeks of the experiment, the animals were euthanized by an overdose of sodium pentobarbital (intraperitoneal injection) 100 mg/kg (Anapental 1, Pharma Tech, Egypt). Following the standard technique [30], the submandibular salivary glands were dissected free, cleaned rapidly from any adherent connective tissues and prepared for the TEM^1^ examination to detect cellular ultrastructural changes of salivary glands.

### Results

All rats survived during the experimental period without any complications.

#### The secretory portion

**Fig. 1:**
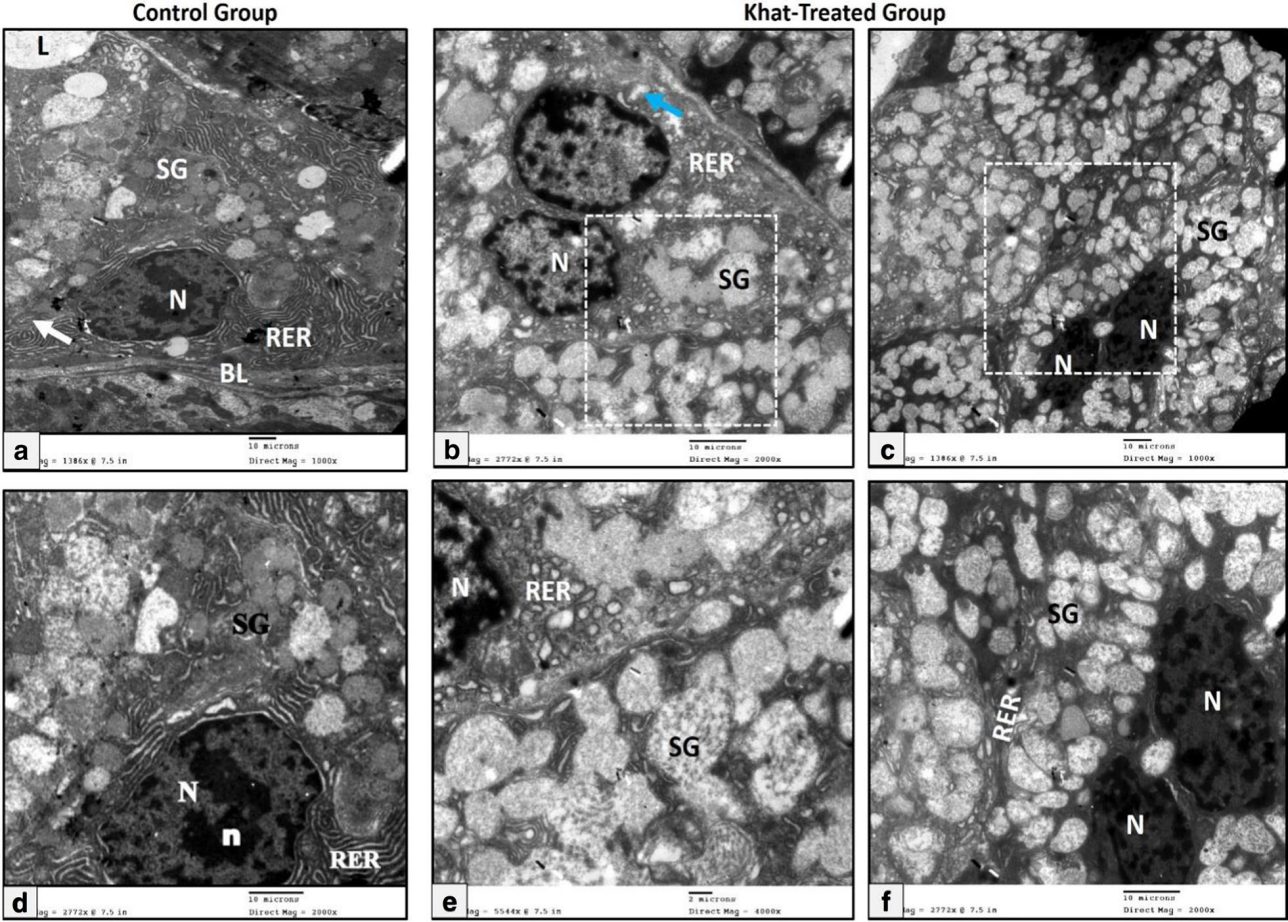
Electron micrographs of the serous cells of the rat submandibular salivary gland showing (**a**) control group: open-faced nucleus (N), well developed rough endoplasmic reticulum (RER), mitochondria (white arrow), basal lamina (BL), variable sized membrane-bounded secretory granules (SG) and lumen (L), **b**, **c** khat-treated group: numerous coalescent electron lucent secretory granules (SG), nucleus with irregular nuclear membrane (N), dilated cisternae of rough endoplasmic reticulum (RER) and swollen mitochondria with degenerated internal cristae (blue arrows) (Uranyl acetate & lead citrate X 1000), **d**–**f** A higher magnification of the squared dotted area (Uranyl acetate & lead citrate X 2000) (Scale bar: 10 µm)

The submandibular salivary glands of the control group revealed the ultrastructure elements of their constituent tissues. The serous secretory cells showed large spherical opened-faced nuclei with electron-dense prominent nucleoli. Parallel cisternae of the rough endoplasmic reticulum (RER) were located basal and lateral to the nucleus. The apical cytoplasm was filled with numerous small membrane-bounded secretory granules of variable electron densities (Fig. 1a).

The khat-treated group revealed moderate changes, such as dilated cisternae of RER and disfigured mitochondria, large number of secretory granules of variable sizes and electron densities. Also, numerous coalescent electron-lucent cytoplasmic vacuoles of variable sizes and densities were observed (Fig. 1b). The nuclei showed some decrease in size with an irregularly delineated nuclear membrane (Fig. 1c).

#### Duct system

**Fig. 2:**
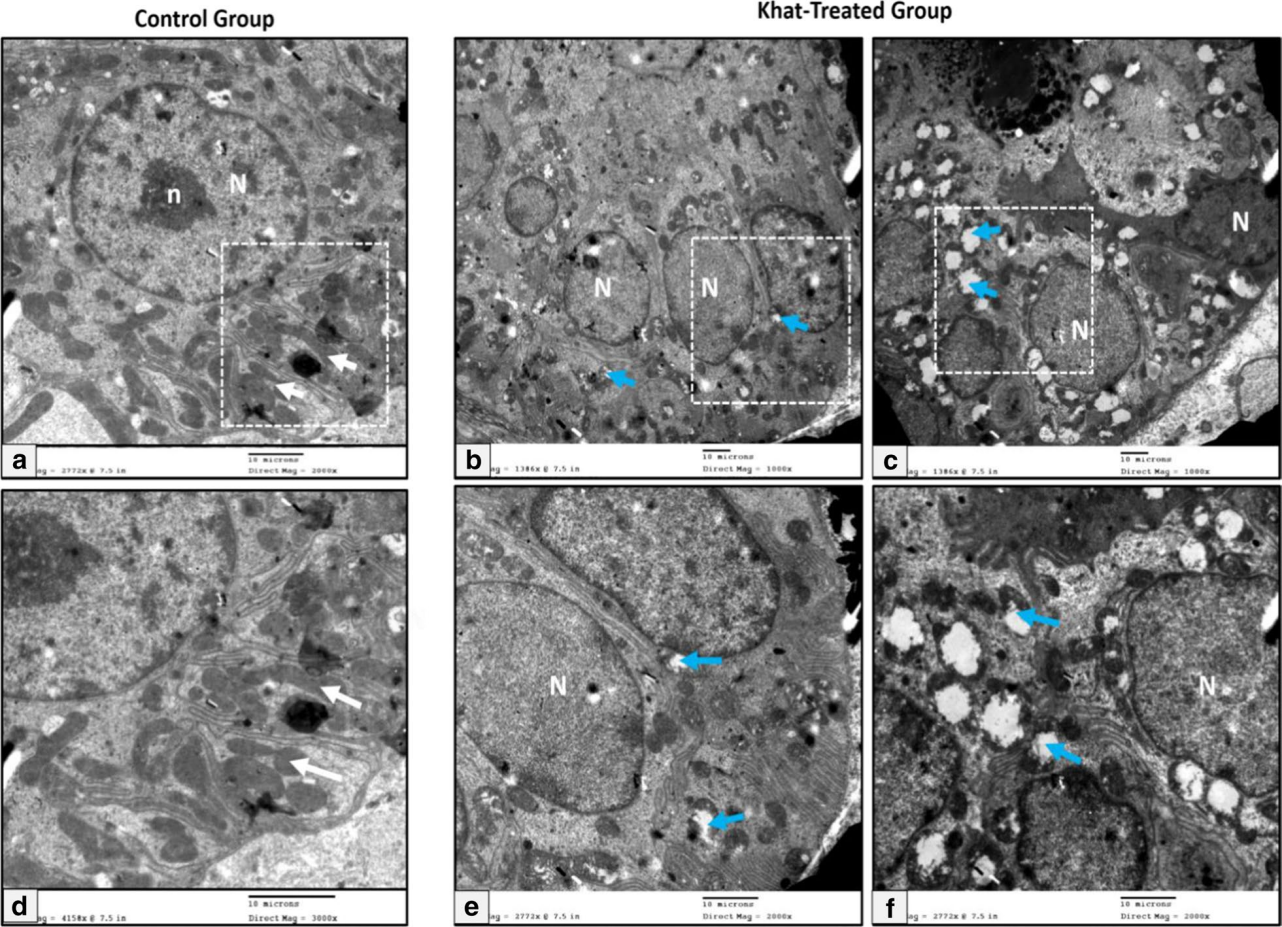
Electron micrographs of the striated duct showing (**a**) control group; characteristic infolded basal membrane containing abundant mitochondria (white arrows), central nucleus (N) with prominent nucleolus (n), **b**, **c** khat- treated group of the submandibular and the sublingual salivary glands respectively; nuclei with variable sizes (N) and basal folding contained degenerated mitochondria (blue arrows) (Uranyl acetate & lead citrate X 1000), **d**–**f** higher magnification of the squared dotted area (Uranyl acetate & lead citrate X 2000) (Scale bar: 10 µm)

Control striated duct cells showed round central open-faced nuclei. The cells were characterized by deep infoldings of their basal plasma membrane, which contained numerous vertically arranged mitochondria arranged parallel to the long axis of the cell. (Fig. 2a). In the khat-treated group, the mitochondria were the most affected cellular organelles in the duct system. They appeared reduced in number and degenerated with loss of the normal architecture of their cristae. The striated ducts showed evident signs of degeneration manifested as a decrease of the basal infoldings associated with markedly swollen and degenerating mitochondria (Fig. 2b, c). Three fields per image were selected randomly and subjected to analysis using cell counter command in the ImageJ software (National Institute of Health (NIH), USA). The results revealed that the average number of damaged mitochondria in the khat-treated group was 7.166 ± 5.49 per field in comparison with the control group 0.766 ± 0.727.

**Fig. 3:**
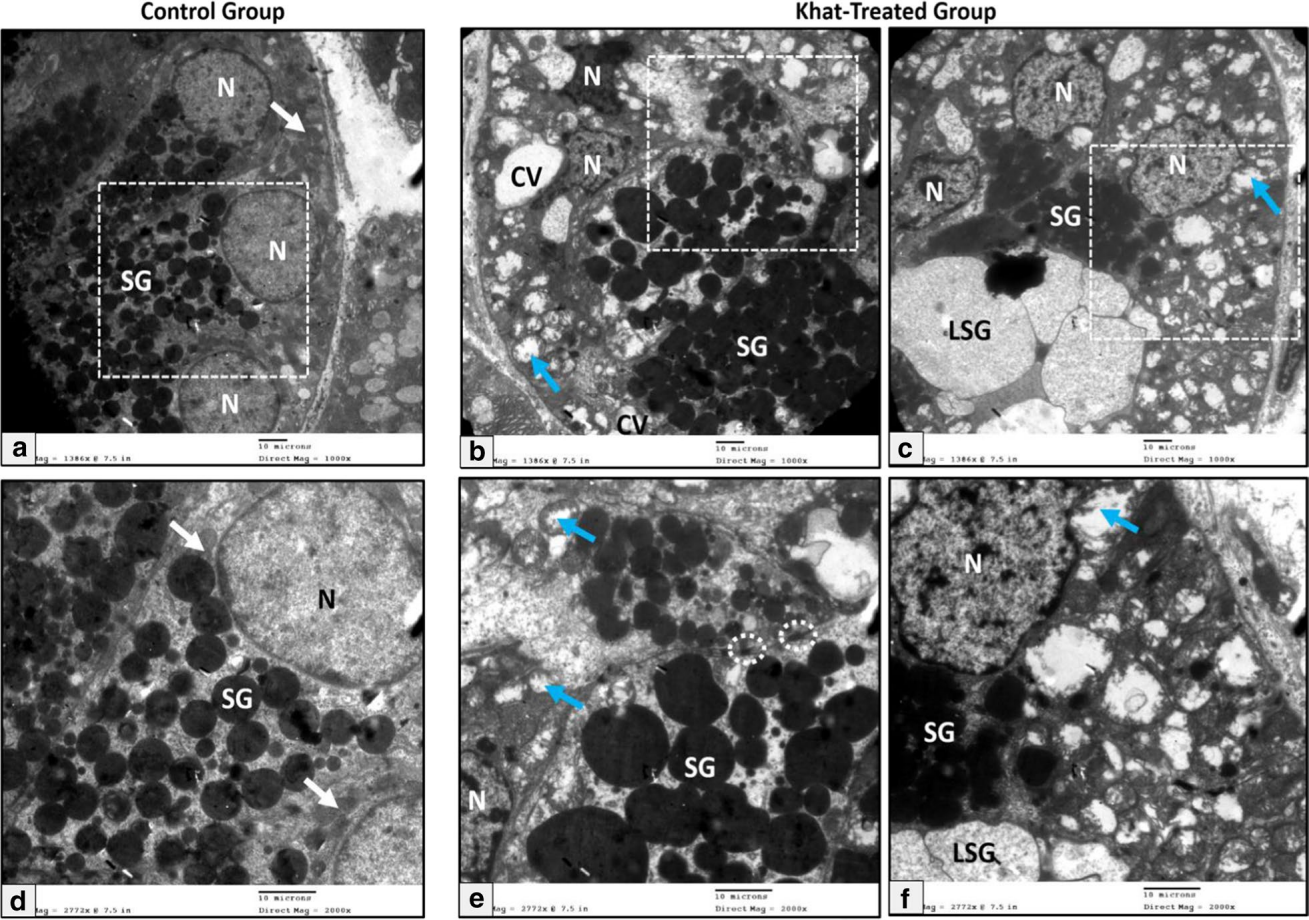
Electron micrographs of the granular convoluted tubules of the rat submandibular salivary gland showing (**a**) control group: basally situated nuclei (N), supranuclearlocalization of numerous electron dense secretory granules of different sizes (SG), mitochondria (white arrows), **b**, **c** khat-treated group: deformed basally situated nuclei (N), electron dense secretory granules (SG), accumulation of electron lucent secretory material (LSG), cytoplasmic vacuoles of variable sizes (CV), degenerated mitochondria (blue arrows) and desmosome junctions between cells (dotted circles) (Uranyl acetate & lead citrate X 1000), **d**–**f** higher magnification of the squared dotted area (Uranyl acetate & lead citrate X 2000) (Scale bar: 10 µm)

The submandibular salivary glands of the rat were characterized by a special type of duct called granular convoluted tubules. The apical part of the cytoplasm of the tall columnar cells of those ducts contained well circumscribed membrane-bounded electron-dense secretory granules. Their open-faced nuclei were located in the basal part of the cells (Fig. 3a). However, the granular convoluted duct cells had wide lumen cast with multiple large electron-lucent secretory materials. In addition to deformed mitochondria, accumulation of electron-lucent secretory material beside the characteristic electron-dense granules of the granular convoluted ducts were observed in the khat-treated group (Fig. 3b, c).

#### Connective tissue stroma

The connective tissue stroma showed collagen fibers, fibroblasts, and blood vessels lined by endothelial cells and filled with electron-dense corpuscle. In addition to the inflammatory cells and fibroblasts, dilated thick-walled blood vessels engorged with electron-dense RBCs were observed in the khat-treated group (See Additional file 1).

### Discussion

Khat produced side effects on the general and oral health of khat chewers as reviewed earlier. This study indicates that Khat extract adversely affected the submandibular salivary glands to different degrees in terms of ultrastructure evaluation. The dryness of the mouth was the major complaint during and after khat chewing and there was an obvious effect of khat on salivary flow rate after khat chewing [23]. To overcome this problem, the chewers usually drink a considerable amount of water or mineral juices during chewing session. This situation might be due to sympathomimetic effects of the active component of khat (cathinone) or due to over-secretion of saliva during the continuous chewing. The salivary dysfunction was in accordance with signs of degeneration observed in this study.

The nuclei of secretory cells exhibited variation in shape and size "pleomorphism", chromatin changes and irregularity of the nuclear membrane. These changes may be due to the decreased metabolic activity and reduction in protein synthesis due to the toxic effect of khat. Along the same line, a molecular-based comparison indicated that khat chewers had a significantly higher micronuclei frequency than non-chewers [31]. Micronucleus frequency in the exfoliated buccal cells exhibited deformed nucleus (irregular in shape and had loops) seen only in khat chewers and smokers group [32]. This might be indicative of aberrations at the cellular level. In accordance with the current findings, the khat extract induced significant morphological changes in human acute myeloid leukemia cells such as cell shrinkage, segregation of intracellular organelles, the formation of membrane blebs or buds, the appearance of cytoplasmic vacuoles, disruption of the nuclear membrane, and condensation and fragmentation of nuclear chromatin [33].

Both secretory and duct cells showed degenerated mitochondria, which occur as a consequence of impairment of metabolic process and are considered as an indication of decreased activity of the duct cells. These changes can be explained by the presence of (−) cathinone, the active compound of khat, that causes damage in the cell membrane integrity, a decline in adenosine triphosphate (ATP levels), and increased mitochondrial superoxide concentrations [34].To some extent, these results were compatible with a study conducted by Lukandu et al. [35], Khat-exposed human oral keratinocytes and fibroblasts showed a swift and sustained decrease of the mitochondrial inner transmembrane potential and associated with a significant decrease in cell survival.

Similarly, Khat produced inner mitochondrial membrane damage, cristae degradation, chromatin condensation, and morphological features of autophagy. Numerous RER showed dilated cisternae, which might be a sign of cell injury rather than a sign of increased secretory activity. The expansion of RER maybe an indicator of disturbances in the exocytosis process of the secretory material or occur as a form of reversible cell injury, leading to the accumulation of secretory materials inside cisternae [36].

Khat treated groups exhibited dilated blood vessels studded with blood corpuscles and other blood vessels showed swollen endothelial lining that caused the collapse of their lumen and obstruction of blood flow. These findings were consistent with the congestion of the central hepatic vein, hypertrophied glomerular capillaries and dilated Bowman’s capsule of kidney demonstrated in animals treated with khat, which may be induced by low- frequency stimulation of cathinone [37, 38]. Overall, the khat extract induced moderate ultrastructure changes in the submandibular salivary glands.

## Conclusion

Oral administration of khat extract produced a range of cellular degenerative alterations in the rats' submandibular salivary glands, including irregular nuclear membrane, mitochondria degeneration and cytoplasmic vacuoles.

## Limitations

This study has a potential limitation in terms of lacking the flow rate assessment. Moreover, further studies are required to determine the molecular profile associated with these changes. In particular, the expression of genes responsible for regulating saliva secretion.

## Supplementary Information

**Additional file 1: Figure S1.** A photograph shows the leaves of khat.** Figure S2.** Transmission electron microscopy images of the mucous cells showing (a) control group: open-faced nucleus (N), rough endoplasmic reticulum (RER), numerous electron lucent secretory granules (Mucin granules) (SG) and basal lamina (BL), (b, c) khat-treated group: nuclei with irregular nuclear membrane (N), dilated cisternae of rough endoplasmic reticulum (RER), swollen degenerated mitochondria (blue arrow), cytoplasmic vacuoles of variable sizes (CV) and secretory granules with disrupted membrane or fused electron lucent material replacing cell cytoplasm (SG) (Uranyl acetate & lead citrate X 1000), (d,e,f) A higher magnification of the squared dotted area (Uranyl acetate & lead citrate X 2000) (Scale bar: 10 µm). **Figure S3.** Transmission electron microscopy images of the connective tissue septa (a,b) control group: blood vessel (BV), fibroblast (F), cross-banding of collagen fibers (CF), (c-f) khat- treated group: thick walled blood vessel with electron dense RBCs (BV), lymphocyte (L) and fibroblast (F) (Uranyl acetate & lead citrate X 2000) (Scale bar: 10 µm).

## Data Availability

All data generated or analysed during this study are included in this published article.

## Footnotes

1 JEOL JEM-100S, JEOL Ltd. Tokyo, Japan with accelerating voltage from 80 to 200 kV and photographed using a charge-coupled device (CCD) camera (Advanced Microscopy Techniques Corp., USA).
