# Supplementary material for: Qualitative ultrastructural analysis of the submandibular salivary glands after administration of khat: in vivo study
Source: BMC Res Notes. 2021 May 13;14:180. doi: 10.1186/s13104-021-05595-8 (PMC8120822; doi:10.1186/s13104-021-05595-8)
Supplement: Supplementary file 1 — Additional file 1: Figure S1. A photograph shows the leaves of khat. Figure S2. Transmission electron microscopy images of the mucous cells showing (a) control group: open-faced nucleus (N), rough endoplasmic reticulum (RER), numerous electron lucent secretory granules (Mucin granules) (SG) and basal lamina (BL), (b, c) khat-treated group: nuclei with irregular nuclear membrane (N), dilated cisternae of rough endoplasmic reticulum (RER), swollen degenerated mitochondria (blue arrow), cytoplasmic vacuoles of variable sizes (CV) and secretory granules with disrupted membrane or fused electron lucent material replacing cell cytoplasm (SG) (Uranyl acetate & lead citrate X 1000), (d,e,f) A higher magnification of the squared dotted area (Uranyl acetate & lead citrate X 2000) (Scale bar: 10 µm). Figure S3. Transmission electron microscopy images of the connective tissue septa (a,b) control group: blood vessel (BV), fibroblast (F), cross-banding of collagen fibers (CF), (c-f) khat- treated group: thick walled blood vessel with electron dense RBCs (BV), lymphocyte (L) and fibroblast (F) (Uranyl acetate & lead citrate X 2000) (Scale bar: 10 µm). [file 13104_2021_5595_MOESM1_ESM.pdf]

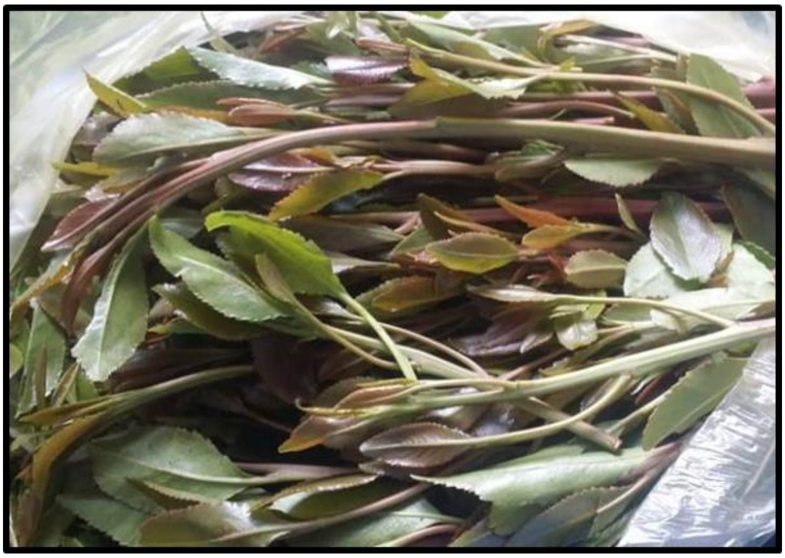

Figure 1: A photograph shows the leaves of khat.

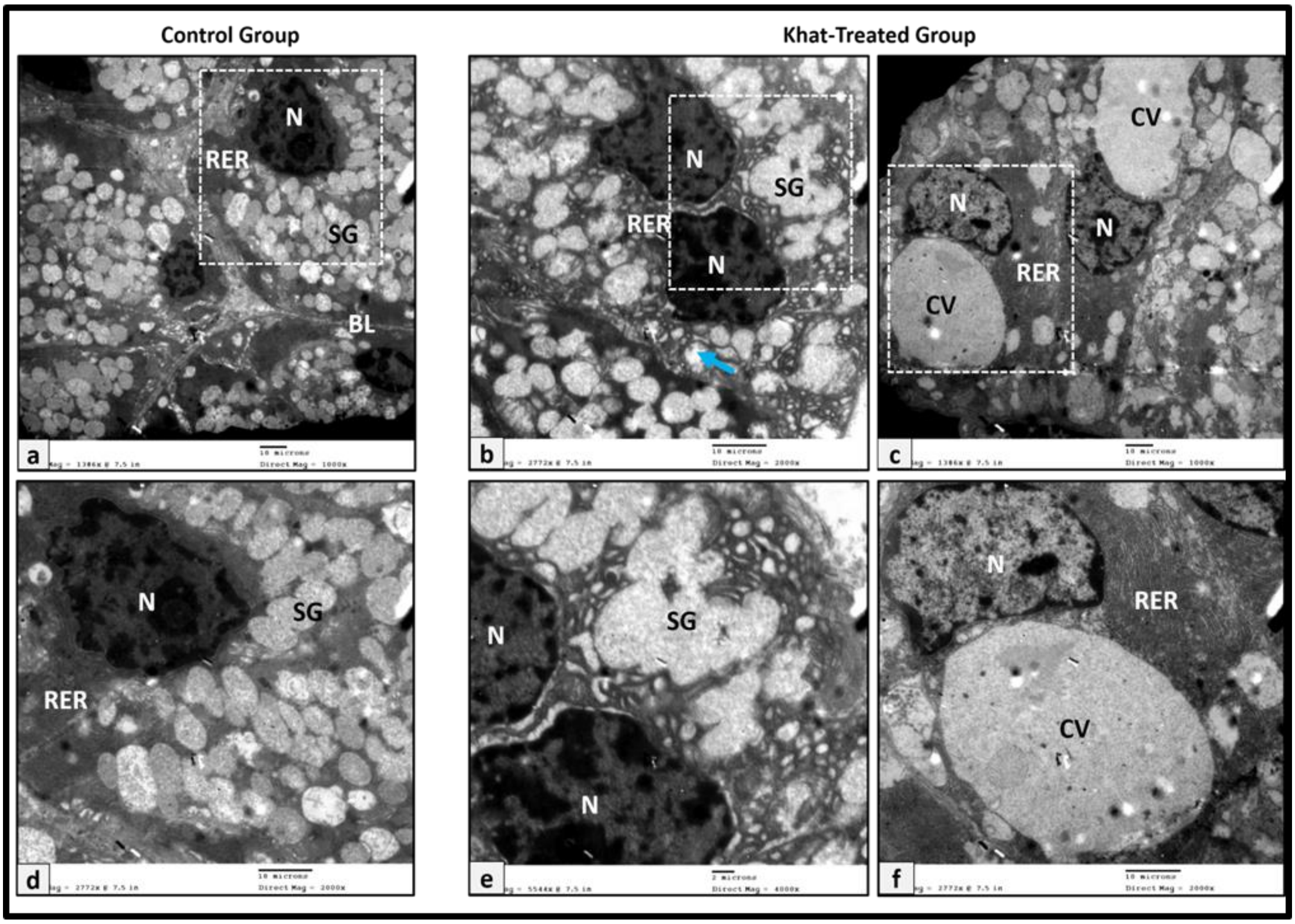

Figure 2: Transmission electron microscopy images of the mucous cells showing (a) control group: open-faced nucleus (N), rough endoplasmic reticulum (RER), numerous electron lucent secretory granules (Mucin granules) (SG) and basal lamina (BL), (b, c) khat-treated group: nuclei with irregular nuclear membrane (N), dilated cisternae of rough endoplasmic reticulum (RER), swollen degenerated mitochondria (blue arrow), cytoplasmic vacuoles of variable sizes (CV) and secretory granules with disrupted membrane or fused electron lucent material replacing cell cytoplasm (SG) (Uranyl acetate & lead citrate X 1000), (d,e,f) A higher magnification of the squared dotted area (Uranyl acetate & lead citrate X 2000) (Scale bar: 10□m).

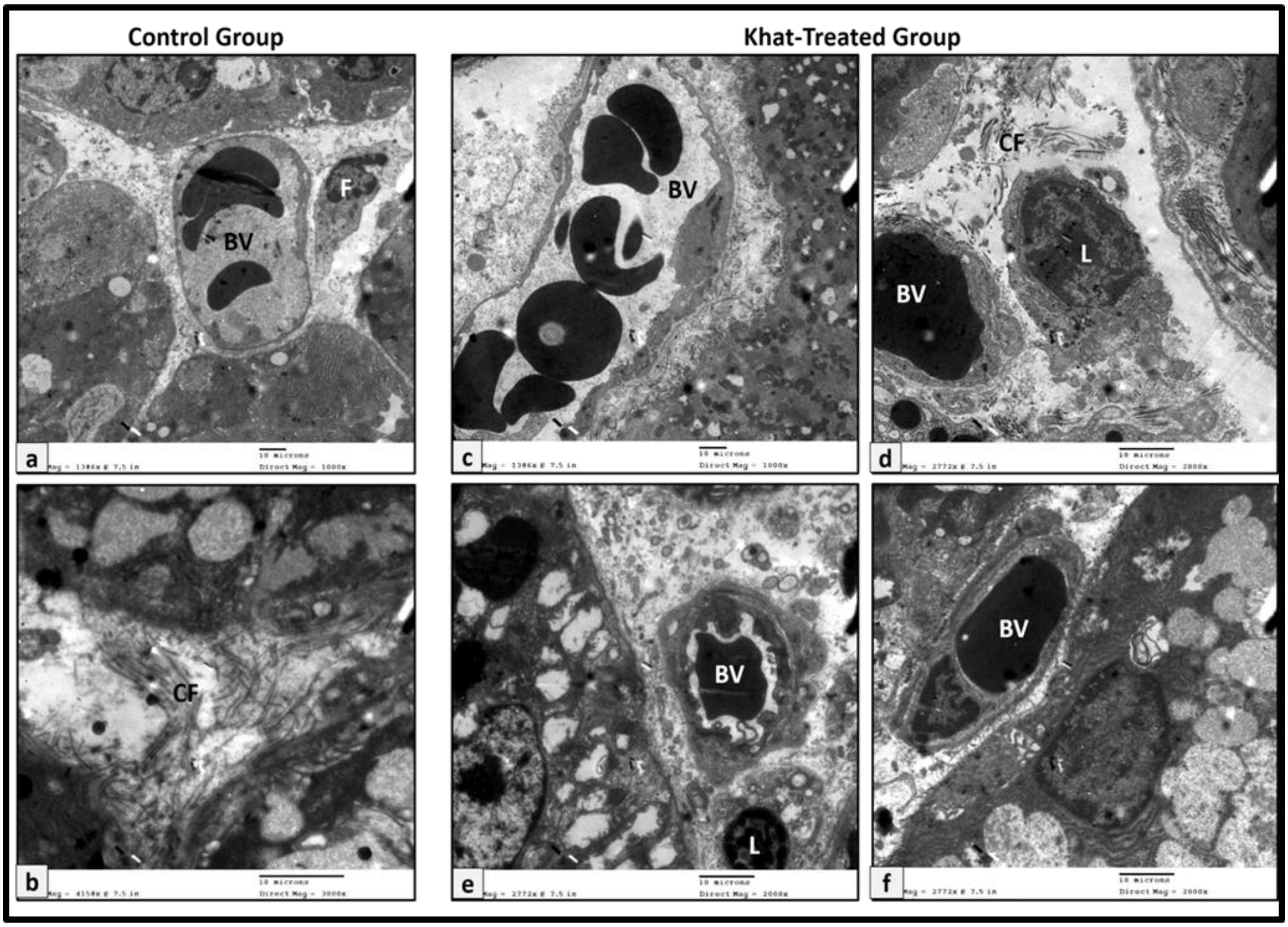

Figure 3: Transmission electron microscopy images of the connective tissue septa (a,b) control group: blood vessel (BV), fibroblast (F), cross-banding of collagen fibers (CF), (c-f) khat- treated group: thick walled blood vessel with electron dense RBCs (BV), lymphocyte (L) and fibroblast (F) (Uranyl acetate & lead citrate X 2000) (Scale bar: 10□m).
